# Xenotransplantation: A New Era

**DOI:** 10.3389/fimmu.2022.900594

**Published:** 2022-06-09

**Authors:** Amber N. Carrier, Anjali Verma, Muhammad Mohiuddin, Manuel Pascual, Yannick D. Muller, Alban Longchamp, Chandra Bhati, Leo H. Buhler, Daniel G. Maluf, Raphael P. H. Meier

**Affiliations:** ^1^ Department of Surgery, University of Maryland School of Medicine, Baltimore, MD, United States; ^2^ Department of Vascular Surgery, Centre Hospitalier Universitaire Vaudois and University of Lausanne, Lausanne, Switzerland; ^3^ Division of Immunology and Allergy, Centre Hospitalier Universitaire Vaudois and University of Lausanne, Lausanne, Switzerland; ^4^ Faculty of Science and Medicine, Section of Medicine, University of Fribourg, Fribourg, Switzerland

**Keywords:** xenotransplantation, kidney, heart, rejection, clinical trial, pig, xenograft

## Abstract

Organ allotransplantation has now reached an impassable ceiling inherent to the limited supply of human donor organs. In the United States, there are currently over 100,000 individuals on the national transplant waiting list awaiting a kidney, heart, and/or liver transplant. This is in contrast with only a fraction of them receiving a living or deceased donor allograft. Given the morbidity, mortality, costs, or absence of supportive treatments, xenotransplant has the potential to address the critical shortage in organ grafts. Last decade research efforts focused on creation of donor organs from pigs with various genes edited out using CRISPR technologies and utilizing non-human primates for trial. Three groups in the United States have recently moved forward with trials in human subjects and obtained initial successful results with pig-to-human heart and kidney xenotransplantation. This review serves as a brief discussion of the recent progress in xenotransplantation research, particularly as it concerns utilization of porcine heart, renal, and liver xenografts in clinical practice.

## Introduction

The limited supply of donor organs and tissues remains the greatest barrier for expanding transplantation, despite many advances in the field over the past several decades. In the United States, there are currently over 100,000 individuals on the national transplant waiting list. Greater than 91,000 of these individuals – approximately 83% – is awaiting a kidney transplant, 3% awaiting a heart transplant, and 10% awaiting a liver transplant (1). This is in contrast with the 22,817 kidney transplants, 3,658 heart transplants, and 8,906 liver transplants performed in 2020 utilizing both living and deceased donor allografts. Worldwide, in 2020 over 129,000 organs were transplanted, which was actually a decrease of 17.6% from the previous year (2). Less than a quarter of these were from living donors. Kidneys and livers donated from living donors represented approximately 28% of the total worldwide organ transplants in 2020. Given the morbidity and mortality of hemodialysis (40-50% survival rate at five years), the generally poor prognosis of patients with end-stage heart or liver failure, and the overall significant cost to the healthcare system that sustaining a patient with end-stage organ failure represents, any advancement that could shorten international wait list times would significantly improve patient health, lifespan, and system expenditures (3). This problem is particularly pronounced in the developing world, where access to hemodialysis or ventricular assist devices (VAD) is often cost-prohibitive and limited, leading to high mortality rates from kidney and heart diseases (4). Artificial liver replacement does not yet exist, and the lack of liver grafts is a global problem.

Xenotransplantation has the potential for reducing the shortage of access to critically needed organ grafts. Animal donor organs and tissue have been subjects of study since the 1960s, and some xenotransplant tissues, particularly heart valves, have been commonly utilized in clinical practice. However, these structures are frequently decellularized extracellular products and therefore do not trigger a robust immune response (5). Until very recently, most research efforts in xenotransplantation focused on creation of donor organs from pigs with various genes edited out using CRISPR technologies and transgenes edited in and utilizing non-human primates for trial. Three groups in the US – University of Maryland, Baltimore (UMB) (6), New York University Langone Health (NYU) (7), University of Alabama at Birmingham (UAB) (8, 9) – have recently moved forward with trials in human subjects. These include one life-sustaining heart xenotransplant in a patient with end-stage heart failure (UMB) (6), and two institutions who have performed kidney xenotransplants in brain-dead subjects (NYU (7) and UAB (8, 9)). Lessons learned so far from these initial clinical xenotransplants will be discussed in this review. Further trials for longer periods of time may be justified in patients for whom dialysis or VAD is either cost-prohibitive or unavailable, or who may not be appropriate candidates for a human allograft (3). This review serves as a brief discussion of the recent progress in xenotransplantation research, particularly as it concerns utilization of porcine heart, renal, and liver xenografts in clinical practice.

## The Development of Suitable Porcine Xenografts

Early research efforts in xenotransplantation focused on utilizing donor organs from non-human primates (NHPs). Despite their close phylogenic relationship with humans, NHPs were found to not be suitable for a number of reasons, including ethical concerns, costs, difficulties in generating genetic modifications, and biosafety (5). Since the 1990s, utilization of donor xenografts from pigs has been the main focus of study. Xenotransplantation utilizing pig xenografts could provide a theoretically endless supply of alternative allografts (10); however, a number of barriers exist for the use of porcine xenografts in clinical practice.

The use of knockout pigs as a source for xenografts has several distinct advantages. Pigs are comparatively straightforward to raise, mature quickly, and will have organs similar in size to a human adult in approximately six months (5, 11, 12). Pigs also reach reproductive maturity rather quickly for large mammals, have relatively large litter sizes, and have physiologic and anatomical similarity to humans (3, 5). For these reasons, pigs were identified as a possible source of renal xenografts and research efforts have focused on transplanting porcine kidneys into NHPs for pre-clinical evaluation of efficacy and suitability. However, the use of NHPs for pre-clinical evaluation of porcine xenografts into human subjects is challenging despite being the standard model for pre-clinical testing of the primate immune response to porcine xenografts and the effects of new immunosuppression regimens (5). NHPs, particularly old-word monkeys (OWMs), often carry naturally occurring specific preformed antibodies to pig cells that are not always present in human serum (13, 14). NHPs, like pigs, express N-glycolylneuraminic acid (Neu5Gc), but when this is knocked out of TKO pigs at least one new antigen, called the “fourth xenoantigen” is exposed which can lead to a robust immune response that does not adequately mimic a TKO pig-to-human model (15). Also, the use of NHP models is considered ethically complex due to their phylogenetic closeness to humans and they take a significant amount of time to physiologically mature. Additionally, NHPs are a very expensive experimental model system to set up and maintain. However, most researchers agree that results in porcine xenograft-NHP models are a necessary step prior to clinical application (5).

## Advanced Immunosuppression Protocols for Xenotransplant Trials

Recent advancements in the gene-editing techniques and immunosuppressive protocols have made clinical xenotransplantation more applicable. Like allotransplant, a major challenge in successful xenotransplantation is to alleviate the risks of immune rejection of the xenotransplant. Following the transplant, three types of rejection may occur- (i) hyperacute rejection, (ii) acute humoral rejection, and (iii) acute cellular rejection.

Hyperacute rejection (HAR) is a type of humoral rejection which occurs within minutes to few hours of transplant due to preformed antibodies in recipient’s blood (16). These preformed antibodies can recognize the α-Gal (galactose-α1,3-galactose) antigen expressed on porcine endothelial cells of organ, which triggers a chain of complement protein activation resulting in the demolition of graft vasculature and finally graft rejection (17). If the graft survives beyond 24 hours, acute humoral xenograft rejection (AHXR) can destroy the transplanted organ. AHXR occurs due to humoral as well as cellular immune responses and is a common cause of xenograft loss seen amongst multiple trials (18). Two non-Gal antigens, Neu5Gc (N-glycolylneuraminic acid) and SDa blood group, are known to be responsible for AHXR (19–21). In the present immunotherapy protocols, HAR and AHXR can be avoided using plasmapheresis and use of pigs genetically modified for the deletion of α-Gal and the two non-Gal antigens (triple knockout or TKO) (5, 22, 23). Acute cellular xenograft rejection (ACXR), which involves NK cells, macrophages, neutrophils, T-cells and B-Cells, also remains major hurdle in long-term xenograft survival (5). Activation of T-cells is one of the main causes of ACXR (5, 24) and alleviation of the T-cell immune responses is critical in the xenotransplantation. Although deletion of α-Gal antigens and expression of human CRPs in donor pigs have been shown to be associated with reduced T-cells responses, this alone is not sufficient for successful long-term survival of xenotransplants (25, 26).

A successful immunosuppression protocol should involve the combination of agents that can increase the length of transplant and have the least side effects on the recipient. Current immunosuppression therapies consist of (i) plasmapheresis to remove the preformed antibodies against the donor, (ii) targeting T-cells and B-cells to keep them low and less active to avoid immune rejection of transplant, (iii) complement protein inhibitors, (iv) anticoagulants, and (v) anti-inflammatory agents to avoid local trafficking of immune cells to the transplant. Various types of transgenic pigs are available to study xenotransplantation. They have been genetically engineered to prevent the humoral and cellular immune responses, coagulation, and complement mediated rejection (27). Of note, not all classes of immunosuppression can be used together, as concurrent use of certain classes together, particularly calcineurin inhibitors and costimulatory blockers, have been shown to have adverse effects (28). Additionally, intravenous immunoglobulin, which is frequently used in the treatment or prevention of rejection, may possibly infuse xeno-antigen antibodies, and its use should be avoided in clinical trials (29).

**Figure 1:**
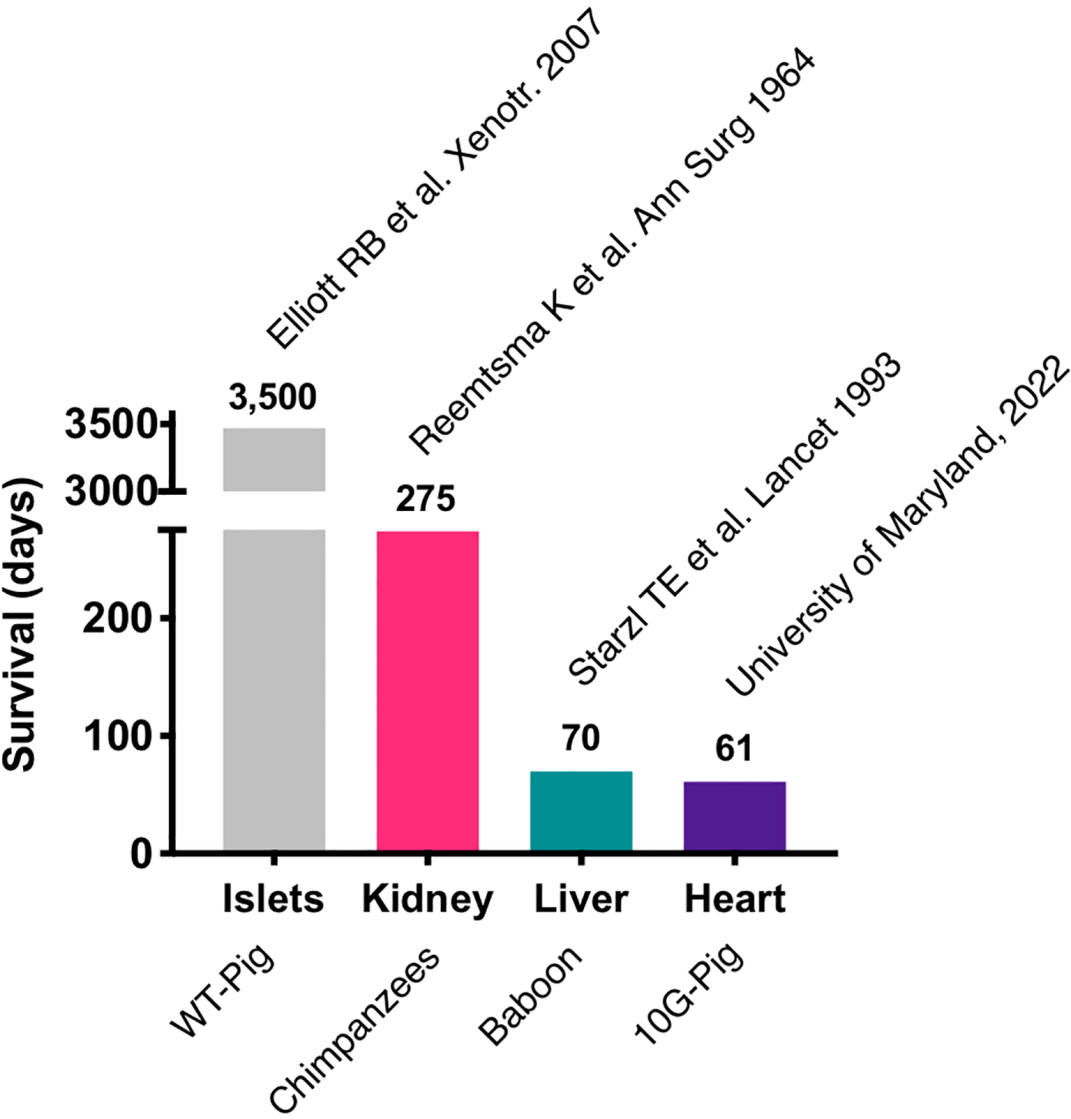
Longest survival of xenotransplanted organs or tissues in human. WT, wild type.

Most of the immunosuppressive therapies that are being tested in pig to NHP xenotransplants block co-stimulatory signals CD40-CD154 and the CD28/CTLA4-CD80/86 interaction, which are required for T-cell activation (30–33). Initial trials with anti-CD154 monoclonal antibodies (mAb) showed promising results in attenuating T-cell response in pig to NHP models (34), but further research showed that anti-CD154mAb had thrombogenic effects and has been discontinued in clinical use (35). Recently, anti-CD40mAb has been shown to be equally effective in blocking the CD40-CD154 interaction. In 2016, Mohiuddin et al. demonstrated that the use of anti-CD40 mAb enhanced the survival of cardiac xenografts up to 945 days in GTKO.hCD46.hTBM pig xenografts in NHPs (33). anti-CD40 mAb also suppress B-cell function by blocking the co-stimulation pathway. In a recent trial of pig to human heart transplant conducted in the University of Maryland, Baltimore, anti-CD40 mAb was used as a part of immunosuppressive regimen along with other immunosuppressants including rituximab (anti-CD20 mAb) and RATG (36, 37). The patient expired 61 days after his cardiac xenotransplant mainly due to non-cardiac causes. The most important lesson learned with this unique case is that the genetically modified pig heart was not subject to hyperacute rejection and functioned appropriately in a human body for about two months. Importantly, these major advances were possible thanks to over two decades of pre-clinical work in large animal models (33, 38–43). This represents the longest survival of a life sustaining pig organ in a human and revived historical xenotransplantation trials, initiated many decades ago using primate organs (**Figure 1**).

In addition to T-cells and B-cells, natural killer (NK) cells also play important role in xenograft rejection (44). Use of genetically modified pigs GalT-KO and HLA-E/human β2 macroglobulin may possibly prevent NK cell-mediated rejection (27, 45). In the past few years, use of regulatory T-cells (Tregs) as immunosuppressive therapy in xenotransplantation has become area of interest. Xenoantigen-specific recipient Tregs can induce donor-specific tolerance by suppressing effector T-cell responses (46, 47). In 2018, one report demonstrated xenograft rejection was correlated with low number of Tregs in peripheral blood lymphocytes in pig to NHPs cardiac xenograft models (48). Wu et al. found that Tregs play an important role in the maintenance of donor-specific tolerance in rodent models of pig neonatal islet xenotransplantation (49). Huang et al. demonstrated that adoptive transfer of *ex vivo* expanded baboon CD39+ Tregs could prevent the porcine islet xenotransplant rejection in primatized NOD-SCID IL-2rγ-/- mice for more than 100 days (50). In addition to regulatory T-cells, Bregs play significant role in transplantation. Bregs can prevent the graft rejection by various mechanisms such as suppressing effector T-cells, activating Tregs, suppressing antigen presentation by dendritic cells and macrophages (51). Another class of regulatory immune cells is tolerogenic dendritic cells (DCs), which can induce central and peripheral tolerance via clonal deletion, activating Tregs and suppressing memory T-cell responses (52). Madelon et al. proved that co-transplantation of autologous IL-10 treated murine tolerogenic DCs enhanced the rat islet xenograft survival in diabetic mice (53). One group in 2018 demonstrated that NHP derived tolerogenic DCs could induce the porcine-specific Tregs (54).

Though an approach combining genetically modified pigs with advanced immunosuppression protocols in NHPs has made the clinical use of pig to human xenotransplant possible in near future, a better understanding of cellular immune responses due to other cells such as NK cells, dendritic cells, and innate cells is required to design an effective immunosuppression protocol.

## Functional and Metabolic Capacities of Pig Xenografts

Though most functions are similar between human allografts and their xenograft counterparts, there are some notable differences in functional and metabolic capabilities that must be accounted for. Though Leo Loeb’s theory regarding protein differences produced by genetically distinct species accounting for graft failure failed to recognize rejection as the ultimate etiology of xenograft failure, it did raise questions about how physiologic differences between species, including their genetic protein structures and subsequent functions, could lead to graft incompatibility (**Table 1**) (55). Porcine lung, kidney, heart, and pancreas allografts have been shown to sustain life in NHPs while porcine liver xenografts have frequently encountered life-threatening complications, implying that these physiologic differences may vary between organs. One important physiologic change triggered by xenografts appears to be thrombotic microangiopathy and systemic consumptive coagulopathy (36), which can be overcome by utilizing pigs that overexpress human coagulation regulation proteins (5).

**Table 1:** Potential physiologic incompatibility between xenograft and recipient.

Organ	Potential incompatibility
**Kidney**	Electrolyte differences (primarily calcium, phosphorus, and potassium) leading to imbalances in recipient Erythropoietin and renin structure dissimilarity between porcine donors and recipients causing anemia and hypoaldosteronism Protein wasting in renal xenografts contributing to hypoalbuminemia and graft loss Hypertensive nephrosclerosis due to higher blood pressures in recipient than in porcine donor
**Heart**	Maladaptive hypertrophy in porcine xenografts resulting in diastolic heart failure Higher blood pressures in NHP/human recipient resulting in xenograft hypertrophy
**Liver**	Lower circulating albumin in porcine donors leading to decreased production and hypoalbuminemia in recipient Coagulopathy, thrombotic microangiopathy, and subsequent graft loss due to uncontrolled activation of the coagulation cascade and contributing to severe thrombocytopenia Potential amino acid differences in protein production leading to changes in function and functional deficiency

In both humans and pigs, kidneys must clear creatinine and other waste products, regulate volume, and accommodate similar volumes of blood flow (56, 57). Pigs tend to maintain somewhat higher levels of potassium, phosphorus, and calcium than do humans and NHPs, whereas albumin and total protein levels are lower. Iwase et al. noted dehydration and hypovolemia with transient increases in serum creatinine in multiple NHP recipients of porcine allografts (56). The recipient NHPs did not exhibit behaviors consistent with dehydration such as changes in oral intake, urine output, body weight, or mental status though other signs of hypovolemia, such as low central venous pressure and visibly dehydrated skin and tissues were present (56). This may be due to molecular differences between porcine and human/NHP renin, as porcine renin has failed to cleave human or NHP angiotensinogen in *in-vitro* models (58, 59). It is suspected that an alternative mechanism for fluid regulation must exist given the maintenance of body weight and overall fluid balance in NHP recipients in prior studies.

Severe proteinuria and hypoalbuminemia were noted in early porcine-NHP xenotransplants, necessitating frequent administration of intravenous albumin to maintain protein balance in an appropriate range (56, 58). The level of proteinuria in these studies was consistent with nephrotic syndrome. However, with recent genetic modifications of donor pigs and improved pharmacological intervention, more recent studies have frequently shown only minimal or modest proteinuria as well as prolonged graft survival, implying that this protein wasting may be a sign of chronic rejection and increased glomerular permeability secondary to podocyte effacement. Proteinuria does not appear to be a normal finding in healthy pigs, though it has been posited as a means by which the porcine kidney lowers albumin levels to those typically found in a pig, which is much lower than that of humans and NHPs (10). Similar to human allograft recipients, immunosuppression regimens including rituximab delay the development of proteinuria in xenotransplant models (58, 60–62).

Porcine hematologic parameters are quite different as well. Pig red blood cell (RBC), white blood cell (WBC), and platelet counts are higher than those of NHPs or humans (56, 57), though overall hemoglobin levels are lower. There is amino acid variability between the erythropoietin produced by pig and NHP/human kidneys, which may contribute to the gradual development of normocytic anemia in NHPs that receive life-sustaining porcine allografts (56) as a molecular incompatibility between porcine erythropoietin and the NHP erythropoietin receptor exists. Repeated blood draws and drug-induced myelosuppression may also contribute to this observation. The administration of recombinant human erythropoietin maintains stable hematocrit levels in NHPs that receive porcine allografts. For potential human xenograft recipients, either the routine administration of recombinant erythropoietin or engineering pigs that produce human erythropoietin may solve this issue.

Cardiac xenotransplants have a different set of physiologic challenges compared to kidneys. First, unaltered cardiac xenografts will undergo maladaptive hypertrophy, leading to diastolic heart failure early after transplantation (63, 64). Utilization of an immunosuppression protocol including temsirolimus and afterload reducing agents can reduce this growth, as this massive cardiac hypertrophy appears to be associated with increased expression of mTOR in cardiac xenografts (64, 65) and the higher blood pressures in primates, including NHPs and humans, can stimulate detrimental cardiac growth for a porcine xenograft (66). This growth can also be eliminated with the use of growth hormone receptor (GHR) knockout xenografts, eliminating the need for medications to overcome the problem (64). An additional change that has been shown to improve survival for porcine xenografts in NHPs has been utilization of non-ischemic heart preservation (5). A similar preservation mechanism was used with the first life sustaining cardiac xenotransplant in a human, performed in January 2022 at the University of Maryland (6).

The significant blood pressure differences between pigs and primates must be addressed as well, even when utilizing knockout xenografts (3). NHPs and humans have systemic vascular resistance (SVR) and mean arterial pressures (MAP) significantly higher than that of age-matched pigs (63), which can provide an extrinsic cause for xenograft hypertrophy. Recipients of porcine xenografts will likely need strict blood pressure control to reduce this extrinsic pressure on the graft that would ultimately lead to failure.

Possible liver xenografts represent a more complex problem. Given the wide and complex array of functions of the liver – including synthesis of most circulating proteins, conjugation and excretion of bilirubin, and detoxification and modification of many incoming chemicals and molecules – the number of potential physiologic incompatibilities between porcine and human/NHP models is very high. Notably, previous hepatic xenotransplant models have been limited by the development of severe thrombocytopenia, coagulopathy, and TMA (5, 67). The addition of exogenous coagulation factors to a co-stimulation blockade in conjunction with the use of α-Gal knockout donor xenografts has allowed for improved success in pig-to-primate liver xenotransplant models, leading to survival times approaching one month in trials (10, 67) with spontaneous platelet recovery and prevention of protein dysregulation. Esker et al. also noted that differences in the amount and activity of various proteins produced by the porcine liver and biliary system – including alkaline phosphatase, lactate dehydrogenase, albumin, and coagulation factors – may also reflect their native species in a xenotransplant and would benefit from genetic alteration to achieve function closer to that of humans.

## Recipient Selection

**Figure 2:**
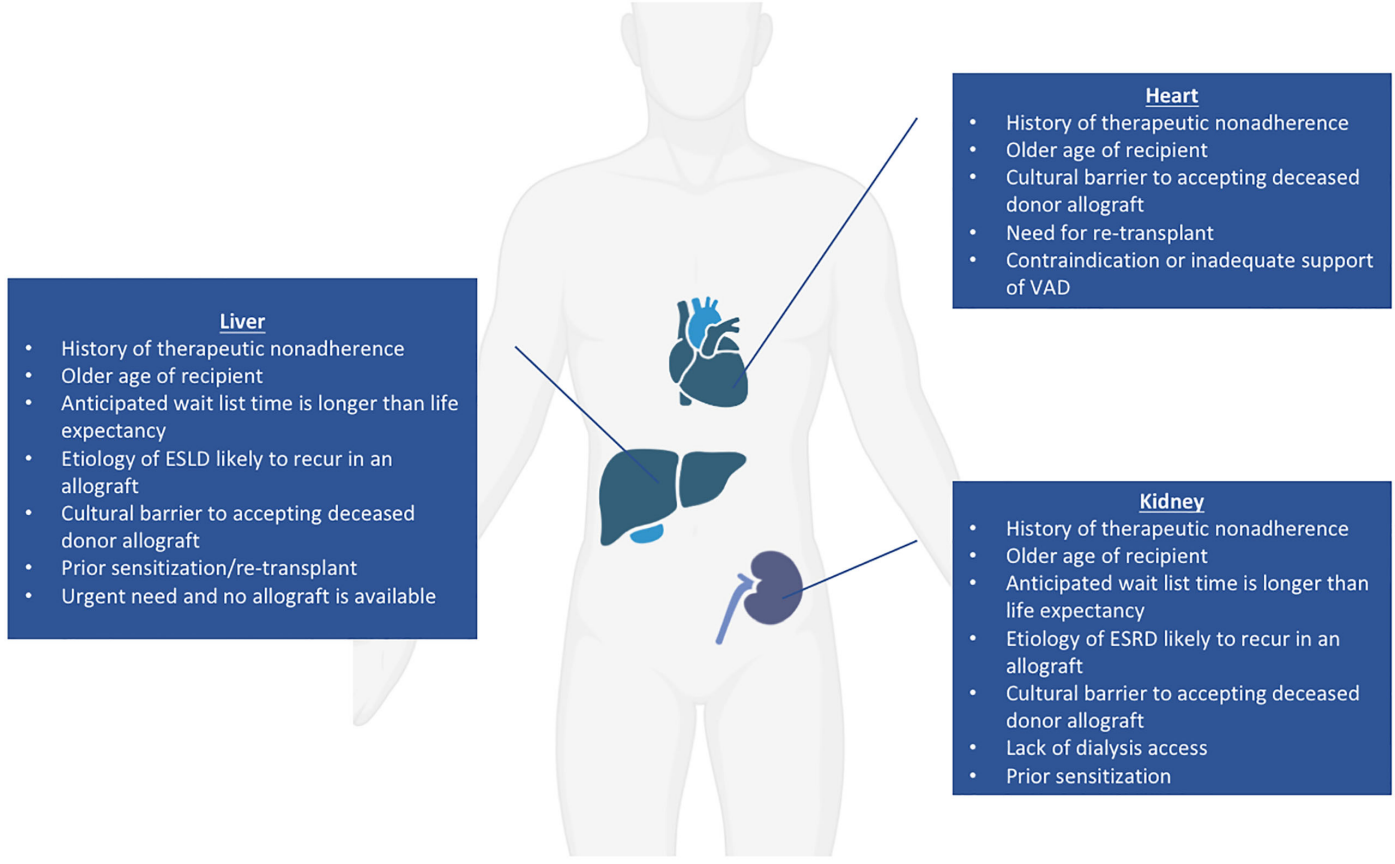
Potential candidates for heart, kidney, and liver xenotransplantation.

Of all organs with the potential for clinical xenotransplant trials, the kidney represents the most straightforward, as it can be easily removed and immunosuppression withdrawn with return to dialysis should the recipient require it (3, 68, 69). The current median wait time for a deceased donor renal transplant is over four years, but approaching ten years in some areas (70); additionally, many patients on the wait list are older than 60, and patients over age 70 are not considered for transplant at some institutions (3). Potential candidates for a xenotransplantation have not been conclusively defined, but general principles regarding good candidates for early xenotransplantation trials have been suggested. Patients for whom their anticipated wait list time is much longer than their life expectancy and who have no identified living donor have been proposed as potential candidates for renal xenotransplantation (3). Those who have renal diseases that are likely to recur in an allograft are also good candidates for xenograft trials as well as those who have exhausted vascular access for hemodialysis but are not candidates for human renal allografts (71). An additional group that may benefit from a xenotransplant would be highly sensitized patients with high titers of anti-human leukocyte antigen (HLA) antibodies where only a limited number of donors can provide a match; anti-HLA antibodies may not react strongly with swine leukocyte antigens (SLA) and therefore not stimulate a significant effect on T-cell and B-cell response (71, 72). Approximately one third to one half of patients on the ESRD waiting list have been shown to have negative crossmatch results when using TKO pigs, implying that those who are difficult to match may benefit from a xenotransplantation trial (15, 73). Alternatively, considering the xenogeneic human TCR repertoire (74), one could consider use of SLA-I knockout pigs in the future (75, 76). However, use of SLA-I depleted organs and cells in transplant raises the concerns about NK cell-mediated injury due to missing self-antigens. This could potentially be avoided by using the transgenic pigs with modified SLA amino acids to prevent the binding of cross-reactive anti-HLA antibodies (77). Patients declined for transplant because of a history of non-compliance might be a category to consider since the risk of wasting a human organ is absent. Society and the medical community might grant an easier access to xenotransplantation in this context. Those for whom deceased human organ donation is culturally taboo may also benefit from a xenotransplant if culturally permissible (78). Kidney xenotransplantation could also be considered in an emergency basis for patients whose life expectancy is short (regardless of the reason) and in whom the continuous need of renal replacement therapy leads to a dramatically decreased quality of life. In summary, potential candidates for a kidney xenotransplantation fall into six main categories: 1) Older age, 2) Sensitized, 3) Lack of dialysis access, 4) Cultural barriers, 5) Non-compliance 6) Short life expectancy with low quality of life (**Figure 2**).

Regarding cardiac xenograft candidates, in addition to the six categories here-above (except for dialysis access), potential candidates could include those who need a re-transplantation, as well as those with contraindications to or who are inadequately supported by the implantation of a ventricular assist device (79). The ideal candidates for liver xenotransplantation could match any of the categories mentioned here above (except for dialysis and VAD access). In addition, the liver has a unique situation since its function cannot be artificially replaced. Consequently, for patients with fulminant, acute, and acute on chronic liver failure, decisions on transplant candidacy need to be made quickly. Thus, acutely ill patients declined for allotransplant unfortunately die within a few days.

Because many patients who are not acceptable candidates for an allogeneic transplantation may be candidates for xenotransplant trials, it will be important to avoid direct comparison of the outcomes between both approaches (3). A fairer comparison will be to initially assess the results of xenotransplantation against the recipient’s anticipated morbidity and mortality without transplantation. Comparison between recipients of xenografts and allografts may still be considered as the science progresses further.

## Recent Xenograft Clinical Trials

On January 7, 2022, University of Maryland, Baltimore reported on the first life-sustaining, 10G-pig xenoheart (80) transplant ever performed in a living human (6). He received a modified immunosuppression protocol including co-stimulation blockade (anti-CD40) maintenance. This transplant was conducted under the umbrella of an emergency New Drug Application (eIND) by the Food and Drug Administration (FDA). This authorization was granted in the setting of an absence of an alternative therapeutic option as the patient was not eligible for an allotransplant nor for a Ventricular Assist Device implantation. Following transplant, the xenograft functioned immediately and ECMO was severed after a few days. The patient was able to be extubated and started the recovery process from his severe deconditioning; the patient had spent several weeks in the hospital prior to transplant. Early results released indicated that the heart was performing extremely well in the absence of rejection. Until day 45-50, he was doing well, despite intermittent infectious episodes. However, in the 8^th^ week post-transplant, his status started to decline, and he unfortunately passed away from multiorgan failure just after reaching the 2-months post-transplant mark. A detailed scientific report of this achievement is currently underwriting, and the lessons learned from this xenotransplant are yet to come. Nonetheless, it clearly appears that hyperacute rejection was defeated and that the xenograft was able to prolong the life of this patient who had no other options. To gain perspective, it is worth noting that the first ever heart allotransplant recipient died 18 days post-transplant of a pneumonia (81). It is also interesting to note that, similarly to the Maryland first heart xenotransplant, the history of the first human heart transplant was also presented to the public long before any scientific publication.

Whether the regulatory process used here, namely an EIND, could be used for a xenogeneic kidney transplant remains to be determined. By definition, the need to urgently replace a kidney is relative as compared to the heart. On the other hand, the kidney presents the advantage of being removed at any time with potentially less severe consequences.

Following this intention to not place a patient under the stress of undergoing a transplant which could immediately fail, it was thought to conduct the initial kidney trial in a human decedent model. On Friday, September 24, 2021, The New York Times reported the results of an experimental pig to human xenotransplantation at New York University led by Dr. Robert Montgomery (7). With consent from the participant’s family, a kidney from an alpha 1,3-galactosyltransferase gene-knockout pig was implanted onto the femoral vessels of a first-person consent organ donor who had progressed into brain death but whose organs were not appropriate for donation. Porcine thymic tissue was implanted under the renal capsule 2 months before the procurement. Over the course of 54 hours, the organ was closely monitored and noted to make urine, clear creatinine, and show no overt signs of rejection (7, 82). A similar procedure was also performed on another brain-dead patient at NYU in late 2021 (83). Several major aspects of the trial, such as the subject’s native renal function, have recently been made available and detailed information about the experiment has just been published in the New England Journal of Medicine (see reference here below). Additionally, there is now data available on inflammatory marker levels, biopsies, and what the patient’s immune response was, and though graft function appears to have been preserved for the duration of the experiment. The experiment also did not utilize a TKO donor kidney, but instead one with a single-gene knockout (⍺-Gal) provided by Revivicor.

On January 20, 2022, the results of a very similar trial performed at UAB were published, using a TKO pig kidney with seven additional genetic modifications (ten genetic modifications or 10G-pigs) transplanted in a brain-dead patient (8, 9). The UAB team noted unequivocally the absence of HAR and documented this with negative flow crossmatches before and after transplant (until 74 hours when the trial was ended) (9). Of note, they used standard immunosuppression with the addition of rituximab, i.e. methylprednisolone taper, anti-thymocyte globulin for a total of 6 mg/kg, and anti-CD20, as well as maintenance consisting of mycophenolate mofetil, tacrolimus, and prednisone. The key learning points from this very initial experience were: 1) no hyper acute rejection, 2) biopsy revealing TMA, 3) urine production but no creatinine clearance. It is unclear if the TMA seen on this patient’s biopsies was secondary to antibody-mediated rejection (AMR) as the subject had some evidence of a hypercoagulable state and inflammation due to their TBI (36). This initial report revealed some limitations inherent to the nature of the recipient, whose physiological state was certainly very distant from a living recipient. The recipient used was a brain-dead donor after bilateral native nephrectomy, and over the course of the study developed multi-organ failure consistent with brain death, including shock liver, disseminated intravascular coagulation, acidemia, and hemorrhagic shock after planned surgical exploration to obtain xenotransplant biopsies on day 3. The pro-inflammatory cytokine storm and hemodynamic instability from these events might have prevented any kidney to function and further enhanced TMA, which was likely preexisting and presumably attributable to the inflammatory-hypercoagulable state caused by traumatic brain injury rather than AMR (36). Other limitations included procurement injuries to the kidneys (a vein injury that required significant clamp time) and the use of a standard immunosuppression in the recipient which might have been insufficient. Other similar trials (7) which will be likely soon reported in a scientific form soon, along with extensive experiments in NHPs (84, 85), seem to indicate that in more stable situations, pig kidney xenografts can clear creatinine. In particular, TMA was avoided in NHPs and long-term xenograft survival was achieved when using the most advanced immunosuppression protocols including co-stimulation blockade (86). For these reasons, leading authors in the filed suggest the next step should be to transplant genetically-engineered pig kidneys into dialysis-dependent patient with no hope of an allotransplant (36).

These recent trials clearly contribute to a significant advance in the field, but also raise a number of new scientific questions. As they were reported in news media, they did have the distinct advantage of drawing public attention to the potential of utilizing xenotransplantation to solve a critical organ shortage (82). The fact that those trials were reported in popular media first reflect the importance, sensitive nature, and fascination generated by xenotransplantation. Due to a concern that the public would see the trials as “unusual” or “unnatural,” there has previously been reticence to report trials involving xenotransplant grafts to media. Public knowledge of this trial and other porcine xenograft trials that have been published in the last several decades will hopefully spark more conversation, research, and public interest (87). On the other hand, the eagerness of the scientific community to see detailed reports of these trials is palpable.

## Ethical Concerns

The use of pigs engineered to grow organs with a low likelihood of rejection raises a number of ethical concerns. Transplant teams utilizing xenografts as a source of donor organs should be prepared to discuss these issues as the regular use of xenografts moves forward in “daily” clinical practice. There are many social benefits that xenotransplantation can help realize, namely relief of the long wait times for a suitable allograft, reduction in dialysis complications, and, especially in some parts of the world, elimination of coercion and financial compensation for organs (71). However, a number of ethical and psychosocial issues exist around xenotransplantation that do not necessarily apply to the use of traditional human allografts.

In some cultures, the use of porcine-based products is considered taboo, though some scholars from these groups will allow for transplants from pigs if the patient would die from organ failure without it (12). There are additionally those who eschew the use of animals and/or animal products as a source of food or dry goods for either religious or animal welfare concerns, and these individuals may take issue with utilization of xenografts as a resource for reducing the transplant wait list (88). There are others still that take issue with raising animals with the exclusive intent of utilizing their organs for xenotransplantation, though the anticipated number of animals needed for this purpose is significantly smaller than the more than 100 million animals killed for food each year in the US alone (82, 88). Keeping in mind the perspective of balancing risks and benefits between use and needs, it is worth mentioning that 240 patients on dialysis die every day in the United States (89).

Pigs raised as a source of xenografts would likely require confinement to reduce the risk of infection and subsequent transmission of an infection to the future recipient of the xenograft. Animals raised under such conditions would not be in an environment in which they would be able to freely roam and interact with other animals like some of their farm-raised counterparts (12, 88). This leads to a conflict among those with animal welfare concerns because the need to raise a xenograft-donor pig in a sterile environment to protect the recipient is in direct conflict with the pig’s natural instincts and needs. The degree of influence of both sides of this conflict has not yet been defined and warrants further exploration involving all stakeholders.

An additional source of ethical concern would be exposing the immunosuppressed patient to the possibility of zoonotic disease transmission. There are some viruses carried by pigs, particularly porcine endogenous retroviruses (PERVs) and Nipah virus that are carried harmlessly by pigs but able to cause significant human disease as human cellular receptors for these viruses exist (88, 90). A PERV is suspected to be the virus responsible for a 2009 epidemic of swine flu that led to the loss of over 250,000 human lives. The risk associated with zoonotic viruses would be amplified by post-transplant immunosuppression (3). There are also some bacteria that may potentially be transmitted by a xenograft that risk horizontal transmission across the community. This is in addition to already an increased infection risk burden that is taken on by transplant recipients because of induction and maintenance immunosuppression. This risk is substantially reduced with raising a pig intended as a xenograft donor in a dedicated sterile, biosecure environment, addressing biosafety concerns but raising the aforementioned ethical issues with regards animal welfare. Specialized molecular assays for viruses may also further reduce this risk (90). Utilizing CRISPR technology, a pig has been produced that has had all PERVs inactivated, reducing the risk of infection with PERVs that could occur with xenotransplantation and allaying this source of ethical quandary (71, 90, 91).

Lastly, public perception of scientific breakthroughs and advancement in the field of xenotransplantation raises both fascination and ethical concerns. There should be general societal involvement in the development of xenotransplant policy, but in the United States public understanding of scientific knowledge and the scientific method is lacking (88). The field of ethics is often considered “outside” of the scope of science, creating a divide between the scientific community and general public on issues of ethics that makes genuine and rational discussion of ethical issues in xenotransplantation challenging. Resolution to this divide would require the integration of both ethical issues and social responsibility into scientific education as well as improvement of science education and understanding in society at large – both of which are noble and challenging goals to achieve.

## Conclusion

Utilization of porcine organs as a source of xenografts has the potential to drastically reduce the long waitlist for transplant and expand eligibility for transplant to those who might otherwise not be candidates. The science behind these trials has advanced considerably and more human clinical trials utilizing porcine xenografts are quickly approaching. However, utilization of xenografts is not without its challenges, and addressing these is critical to both clinical success and public acceptable of early porcine-to-human xenograft trials. Recent media attention around the first clinical trials has cast attention on the field, and this will hopefully continue to stimulate a conversation about the ethical and social concerns regarding the use of porcine xenografts as initial trials are developed, conducted, and reported in a scientific format (92). Ideally, these trials would focus on determining appropriate recipient selection criteria and the identification of an appropriate immunosuppression regimen for xenograft recipients as these are currently substantial unknowns that require further investigation in order for xenotransplants to be integrated into standard clinical practice.

## Author Contributions

AC, AV, MM, MP, YM, AL, CB, LB, DM, and RM designed the study, collected the data, interpreted the data, and wrote the manuscript. AC, AV, MM, MP, YM, AL, CB, LB, DM, and RM had full access to all of the data in the study and take responsibility for the integrity of the data and the accuracy of the data analysis.

## Conflict of Interest

The authors declare that the research was conducted in the absence of any commercial or financial relationships that could be construed as a potential conflict of interest.

## Publisher’s Note

All claims expressed in this article are solely those of the authors and do not necessarily represent those of their affiliated organizations, or those of the publisher, the editors and the reviewers. Any product that may be evaluated in this article, or claim that may be made by its manufacturer, is not guaranteed or endorsed by the publisher.

## Abbreviations

AHXR, Acute humoral xenograft rejection; AMR, Antibody-mediated rejection; CRISPR, Clustered Regularly Interspaced Short Palindromic Repeats; eIND, Emergency New Drug Application; FD, Food and Drug Administration; GHR, Growth hormone receptor; HLA, Human leukocyte antigen; HAR, Hyperacute rejection; mTOR, Mammalian target of rapamycin; MAP, Mean arterial pressures; mAb, Monoclonal antibodies; NYU, New York University Langone Health; NHPs, Non-human primates; OWMs, Old-word monkeys; PERVs, Porcine endogenous retroviruses; RBC, Red blood cell; SLA, Swine leukocyte antigens; SVR, Systemic vascular resistance; TMA, Thrombotic microangiopathy; TKO, Triple knockout or; UAB, University of Alabama at Birmingham; UMB, University of Maryland, Baltimore; VAD, Ventricular assist devices; WBC, White blood cell.
